# Public Health Surveillance of Behavioral Cancer Risk Factors During the COVID-19 Pandemic: Sentiment and Emotion Analysis of Twitter Data

**DOI:** 10.2196/46874

**Published:** 2023-11-02

**Authors:** Nicolette Christodoulakis, Wael Abdelkader, Cynthia Lokker, Michelle Cotterchio, Lauren E Griffith, Leigh M Vanderloo, Laura N Anderson

**Affiliations:** 1 Department of Health Research Methods, Evidence, and Impact, McMaster University Hamilton, ON Canada; 2 Dalla Lana School of Public Health, University of Toronto Toronto, ON Canada; 3 Population Health and Value Based Health Systems, Ontario Health Toronto, ON Canada; 4 ParticipACTION Toronto, ON Canada; 5 School of Occupational Therapy, Western University London, ON Canada

**Keywords:** cancer risk factors, Twitter, sentiment analysis, emotion analysis, social media, physical inactivity, poor nutrition, alcohol, smoking

## Abstract

**Background:**

The COVID-19 pandemic and its associated public health mitigation strategies have dramatically changed patterns of daily life activities worldwide, resulting in unintentional consequences on behavioral risk factors, including smoking, alcohol consumption, poor nutrition, and physical inactivity. The infodemic of social media data may provide novel opportunities for evaluating changes related to behavioral risk factors during the pandemic.

**Objective:**

We explored the feasibility of conducting a sentiment and emotion analysis using Twitter data to evaluate behavioral cancer risk factors (physical inactivity, poor nutrition, alcohol consumption, and smoking) over time during the first year of the COVID-19 pandemic.

**Methods:**

Tweets during 2020 relating to the COVID-19 pandemic and the 4 cancer risk factors were extracted from the George Washington University Libraries Dataverse. Tweets were defined and filtered using keywords to create 4 data sets. We trained and tested a machine learning classifier using a prelabeled Twitter data set. This was applied to determine the sentiment (positive, negative, or neutral) of each tweet. A natural language processing package was used to identify the emotions (anger, anticipation, disgust, fear, joy, sadness, surprise, and trust) based on the words contained in the tweets. Sentiments and emotions for each of the risk factors were evaluated over time and analyzed to identify keywords that emerged.

**Results:**

The sentiment analysis revealed that 56.69% (51,479/90,813) of the tweets about physical activity were positive, 16.4% (14,893/90,813) were negative, and 26.91% (24,441/90,813) were neutral. Similar patterns were observed for nutrition, where 55.44% (27,939/50,396), 15.78% (7950/50,396), and 28.79% (14,507/50,396) of the tweets were positive, negative, and neutral, respectively. For alcohol, the proportions of positive, negative, and neutral tweets were 46.85% (34,897/74,484), 22.9% (17,056/74,484), and 30.25% (22,531/74,484), respectively, and for smoking, they were 41.2% (11,628/28,220), 24.23% (6839/28,220), and 34.56% (9753/28,220), respectively. The sentiments were relatively stable over time. The emotion analysis suggests that the most common emotion expressed across physical activity and nutrition tweets was trust (69,495/320,741, 21.67% and 42,324/176,564, 23.97%, respectively); for alcohol, it was joy (49,147/273,128, 17.99%); and for smoking, it was fear (23,066/110,256, 20.92%). The emotions expressed remained relatively constant over the observed period. An analysis of the most frequent words tweeted revealed further insights into common themes expressed in relation to some of the risk factors and possible sources of bias.

**Conclusions:**

This analysis provided insight into behavioral cancer risk factors as expressed on Twitter during the first year of the COVID-19 pandemic. It was feasible to extract tweets relating to all 4 risk factors, and most tweets had a positive sentiment with varied emotions across the different data sets. Although these results can play a role in promoting public health, a deeper dive via qualitative analysis can be conducted to provide a contextual examination of each tweet.

## Introduction

### Background

The COVID-19 pandemic and its associated public health mitigation strategies have dramatically changed the patterns of daily life activities worldwide. To control the spread of SARS-CoV-2, necessary public health restrictions, including requirements to work from home, limit contact with others, and close schools were implemented in March 2020 [1]. Although these strategies were necessary to control the spread of SARS-CoV-2, there were unintentional adverse consequences on health behaviors [2,3]. Cancer is a leading chronic disease worldwide with many known modifiable risk factors, including smoking, alcohol consumption, poor nutrition, and lack of physical activity [4,5]. International cancer prevention recommendations target these behaviors [4,5]. The Global Burden of Diseases study has estimated that physical inactivity is associated with up to 25% of breast and colon cancer cases, smoking is responsible for 71% of lung cancer cases, low fruit and vegetable intake is responsible for 14% of gastrointestinal cancer deaths, and alcohol consumption is responsible for 30% of esophageal and liver cancer deaths [6]. These behavioral risk factors have a profound impact on cancer incidence and mortality, and many studies have reported decreases in physical activity; increases in sedentary behaviors; and increases in alcohol intake, tobacco use, and junk food consumption during the pandemic [7-12].

Public health restrictions and physical distancing during the COVID-19 pandemic have challenged the traditional methods of data collection, such as in-person surveys and interviews. Furthermore, traditional health surveys for public health surveillance have other limitations, including selection bias, lack of generalizability, high cost, and extended length of time required for data collection. Innovative means of obtaining meaningful and timely community-level data could help improve public health surveillance and provide valuable real-time intelligence to inform interventions during public health emergencies. Gaps in timely data collection to support chronic disease prevention efforts in public health have been identified as a concern [13-17]. Nontraditional data sources such as data collected through social media, web-based surveys, and mobile phone tracking offer new ways of accessing community-level risk factor information quickly, repeatedly, and easily. During the COVID-19 pandemic, the intensity of internet and social media use increased [18]. Social media platforms are places where people share vast amounts of information including for communication with others and the expression of personal opinions [19]. Social media content has increasingly been used for research data, and in combination with advances in natural language processing (NLP), there is the potential to extract more meaningful and accurate information [20]. NLP is a subfield of artificial intelligence and machine learning that uses computers to process and analyze human languages, speech, or text [20]. NLP packages are able to evaluate the content of text-based data such as tweets on both sentiment (positive and negative markers) and emotion data. Given the current infodemic, Twitter (subsequently rebranded as X) data are increasingly being used for research studies because they contain a large sample size owing to the vast number of users, geographical diversity, and public accessibility. Examples of potential applications of NLP in public health include identifying at-risk populations through the analysis of risk behaviors using social media and conducting environmental scans for public health risk assessment [21].

Specific to behavioral risk factors and cancer, previous studies have conducted sentiment analyses of Twitter data on the topics of physical activity and smoking and found that it is feasible to observe some trends over time and may be valuable for real-time public health surveillance [22,23]. Other studies have found a positive correlation between individuals’ tweets and their subsequent actions; for example, those who tweeted positive tobacco messages (in favor of tobacco products or any positive message about any tobacco product) were more likely to use tobacco and another study found that the proportion of alcohol-related Twitter posts was associated with drinking outcomes [24,25]. This highlights the importance of using social media platforms such as Twitter as they provide individual generated real-time data that have the potential to inform public health care practice. Investigating changes in the sentiment and emotion of tweets related to behavioral cancer risk factors during the COVID-19 pandemic will inform the feasibility of using Twitter data for public health surveillance of chronic disease risk factors. Using social media platforms such as Twitter provides a unique opportunity to obtain data that has the potential to inform public health surveillance and practice and may identify novel opportunities for cancer prevention.

### Objectives

The objectives of our study were to explore the feasibility of conducting a sentiment and emotion analysis using Twitter data to evaluate 4 behavioral cancer risk factors (physical inactivity, poor nutrition, alcohol consumption, and smoking) over time during the first year of the COVID-19 pandemic.

## Methods

### Overview

**Figure 1:**
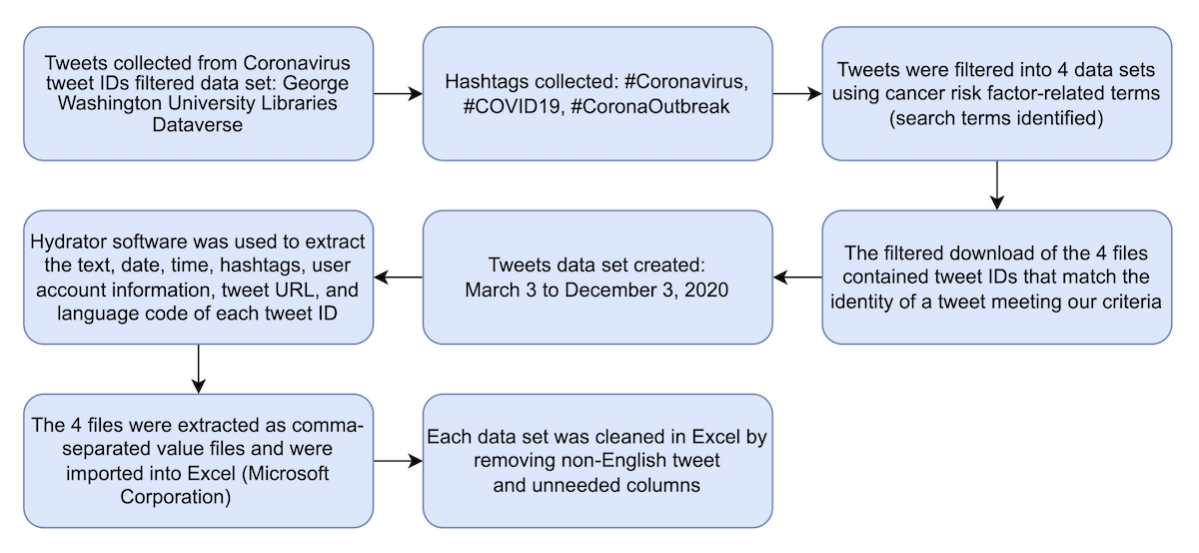
Flowchart illustrating the data collection process.

This study consisted of 5 steps that were incorporated from another published study [26]. The steps include (1) collecting COVID-19 tweets from an established data set, (2) filtering the tweets into 4 data sets using cancer risk factor–related terms, (3) preparing the data set for model training, (4) training a model to predict the sentiment class from negative to positive, and (5) applying NLP to identify emotions expressed in the tweets. The data collection steps are explained in the *Study Design and Data Source* and *Search Strategy and Data Set Creation* sections and illustrated in the flowchart shown in Figure 1.

### Study Design and Data Source

The data were collected from a COVID-19–filtered data set that was made available by the George Washington University Libraries Dataverse, which is a part of the Harvard University Dataverse [27]. This data set contains the tweet IDs of 354,903,485 tweets related to coronavirus or COVID-19. The tweets were collected between March 3, 2020, and December 3, 2020, a time of uncertainty and use of social restrictions globally. Tweets were collected via the Twitter application programming interface (API) using the Social Feed Manager [27]. These tweets were collected using the POST statuses and filter method of the Twitter Streaming API. This method returns public statuses that match one or more filtered search terms. Numerous terms were specified with this method, which permits a single connection to the Streaming API [28]. These tweets were captured worldwide as the Twitter API provides access to tweets from users worldwide.

The hashtags of focus for this study were #Coronavirus, #COVID19, and #CoronaOutbreak [27]. The TweetSets Online platform, created by Littman et al [29] in 2018, was used to apply our specific search terms. Only original tweets were included, and retweets were excluded.

### Search Strategy and Data Set Creation

For each risk factor, a separate tweet data set was created by applying relevant search terms to filter the tweets. On the TweetSets Online platform, the group of search terms for each factor was entered into the “contains one of the terms” category for each data set. This means that the tweets extracted in each of the data sets had to contain at least 1 of the search terms provided. The search terms for each data set were obtained from multiple sources. Some of the search terms were chosen using Medical Subject Headings on MEDLINE. Other search terms were included by conducting a literature review and using the words that other studies used on similar topics [22,25]. However, terms that people could use in a context that was not for its intended purpose in this study were excluded. For example, in the physical activity data set, the word *running* or *run* was not used as its context could be used as *I am running late for work*, which negates the search for physical activity tweets. On the basis of our approach, we created a list of the most relevant search terms. The search terms applied for each risk factor that were used to create the 4 data sets are listed in Table 1.

**Table 1:** Search terms applied for each risk factor to create 4 unique data sets.

Risk factor	Search terms applied
Physical inactivity	“physical activity,” “fitness,” “gym,” “exercise,” “work out,” “treadmill,” “stationary bike,” “free weights,” “rowing machine,” and “elliptical”
Poor nutrition	“fruit,” “vegetable,” “fruits,”, “vegetables,” “nutrition,” “nutritionist,” “vitamin,” “vitamins,” “calories,” and “calorie”
Alcohol consumption	“alcohol,” “booze,” “beer,” “liquor,” “champagne,” “wine,” “moonshine,” “cocktail,” “drunk,” “hung over,” “alcoholic,” “rum,” “alcoholism,” “intoxicated,” “vodka,” and “whiskey”
Smoking	“smoke,” “smoking,” “cigarette,” “vaping,” “nicotine,” “Juul,” “Juul pod,” “vape,” “cig,” “cigars,” “cigar,” “tobacco,” “vape stick,” and “cigarettes”

The output of the TweetSets filtered download was 4 files containing tweet IDs to the identity of a specific tweet that met the criteria of our search term filters. To obtain the text, date, time, hashtags, user account information, tweet URL, and language code (the tweet metadata), Hydrator software was used for each file [30]. Hydrator is an Electron-based application for hydrating Twitter ID data sets [30]. It takes the file of tweet IDs and extracts them as a comma-separated values file. After putting the 4 files into Hydrator and extracting them as comma-separated values files, they were imported into Excel (Microsoft Corporation) for cleaning and analysis. In Excel, we limited the tweets to English language.

### Ethical Considerations

This study did not require ethics approval because all data collected were publicly available. There is no means within this paper or its supporting materials to establish the identification of users and their corresponding tweets.

### Sentiment Analysis

**Figure 2:**
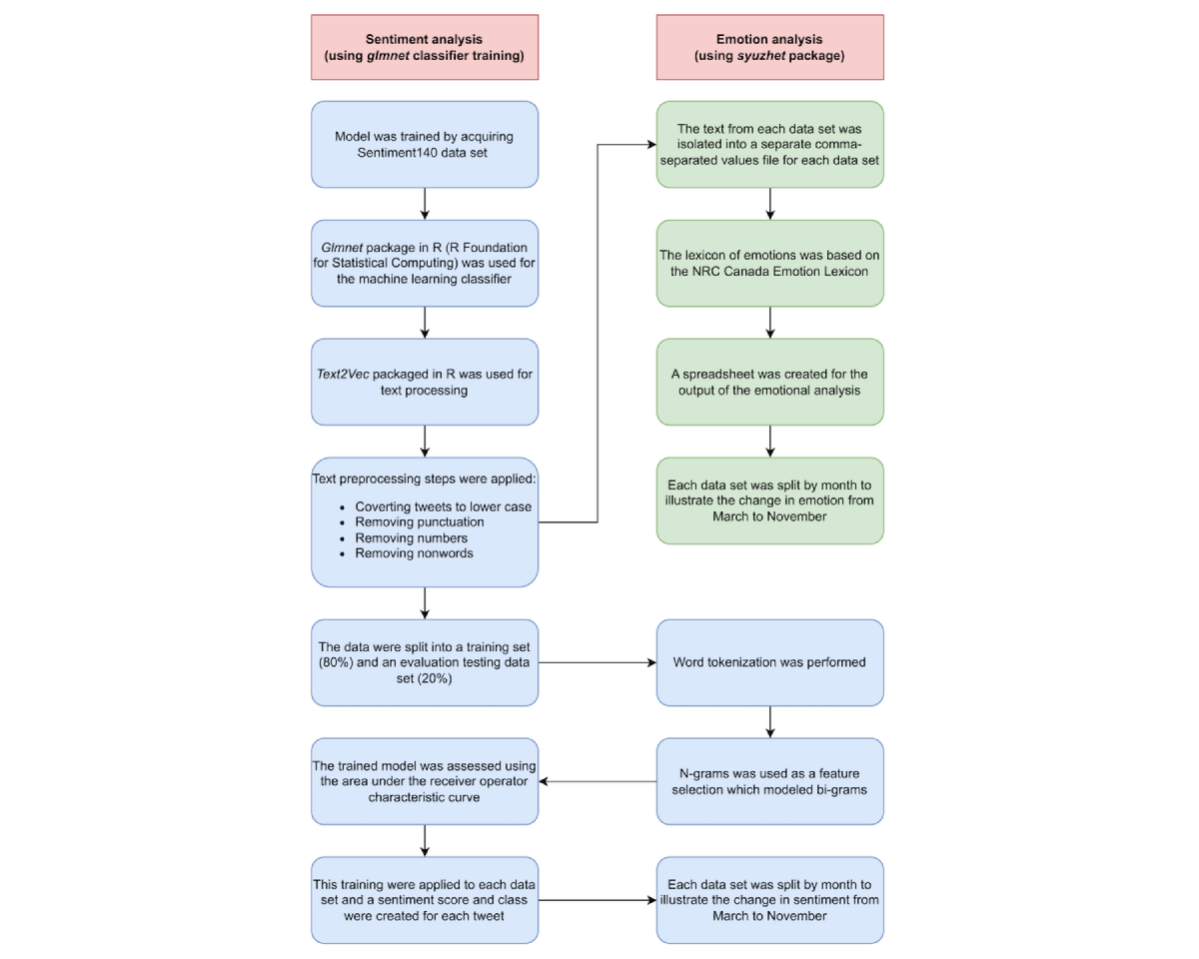
Flowchart depicting both the sentiment and emotion analysis. NRC: National Research Council.

The analysis steps conducted for both the sentiment and emotion analysis are summarized by the flowchart provided in Figure 2.

A supervised machine learning approach was used to load and train the data set. The model was trained using the Sentiment140 training data set, which is a large data set consisting of 1.6 million tweets that have been labeled as positive and negative sentiments based on the emoticons in the tweet’s text [31]. Sentiment analysis is a method of extracting opinion polarity to determine whether the data are positive, negative, or neutral [32]. Sentiment analysis was conducted separately for each of the 4 data sets using the RStudio software (Posit) [33]. We used the *glmnet* package in R (R Foundation for Statistical Computing) for the machine learning classifier and the *Text2Vec* package in R for text processing [34-37].

After acquiring the Sentiment140 training data set created by Go et al [38], text preprocessing steps were applied. This included converting the tweets to lower case and removing punctuation, numbers, and nonwords. The data set was then split into a training set (80% of the data) and an evaluation testing data set (20% of the data set). Word tokenization was performed before continuing the sentiment and emotion analysis. Words in the training data set were tokenized using the *Text2Vec* package to condense and reduce phrases or words into tokens [39]. This process identifies important words in a sentence or a tweet.

N-grams was used as the feature selection, which refers to the process of selecting a subset of relevant features (words, variables, or predictors) to be used in the model construction. An N-gram is the sequence of a given number of words (N). The N-gram model predicts the most probable word that may follow a certain sequence. It is a probabilistic model trained on a corpus of text [40]. In addition, the N-gram model preserves the word locality information. In this study, bigrams (ie, a sequence of 2 words) were used [41,42]. This process consisted of the text having to be turned into an array of numbers for the machine to understand the text within the given data sets [43]. This process is called text vectorization, which uses vectorized bigrams and organizes them in a document-term matrix [26]. A document-term matrix is a mathematical matrix that shows the frequency of terms in a collection of text [44]. The classifier generated probability values for each tweet ranging between 0 and 1; 0 tends toward the most negative sentiment, and 1 tends toward the most positive sentiment. Values between 0.35 and 0.65 are considered neutral [45]. The trained model was applied to the evaluation test data set with performance assessed using the receiver operator characteristic curve and area under the receiver operator characteristic curve. The sentiment classifier used had an area under the receiver operator characteristic curve of 0.894. This demonstrates that the classifier used has a good ability to discern between negative and positive sentiments [26].

### Emotion Analysis

To evaluate the emotion expressed within tweets in the 4 data sets, the *syuzhet* package was used in R to extract and analyze emotion in the text data [46]. The package is based on the theory of narrative emotions and uses a computational linguistic approach to extract the emotional context from the text. It uses various sentiment dictionaries. In this study, the lexicon of emotions based on the National Research Council (NRC) Canada Emotion Lexicon [47] developed by Mohammed [48] was used. The NRC Emotion Lexicon is a set of English words and their associations with 8 basic emotions and 2 sentiments. The 8 emotions that the package could detect included anger, anticipation, disgust, fear, joy, sadness, surprise, and trust [47,49]. The 2 sentiments are negative and positive [47,49].

## Results

### Data Set Results

After the tweets were collected and filtered using the search terms from the coronavirus data set and imported into Excel, the data were cleaned before analysis. There were 90,813 tweets in the physical activity data set, 50,396 tweets in the nutrition data set, 74,484 tweets in the alcohol data set, and 28,220 tweets in the smoking data set.

**Figure 3:**
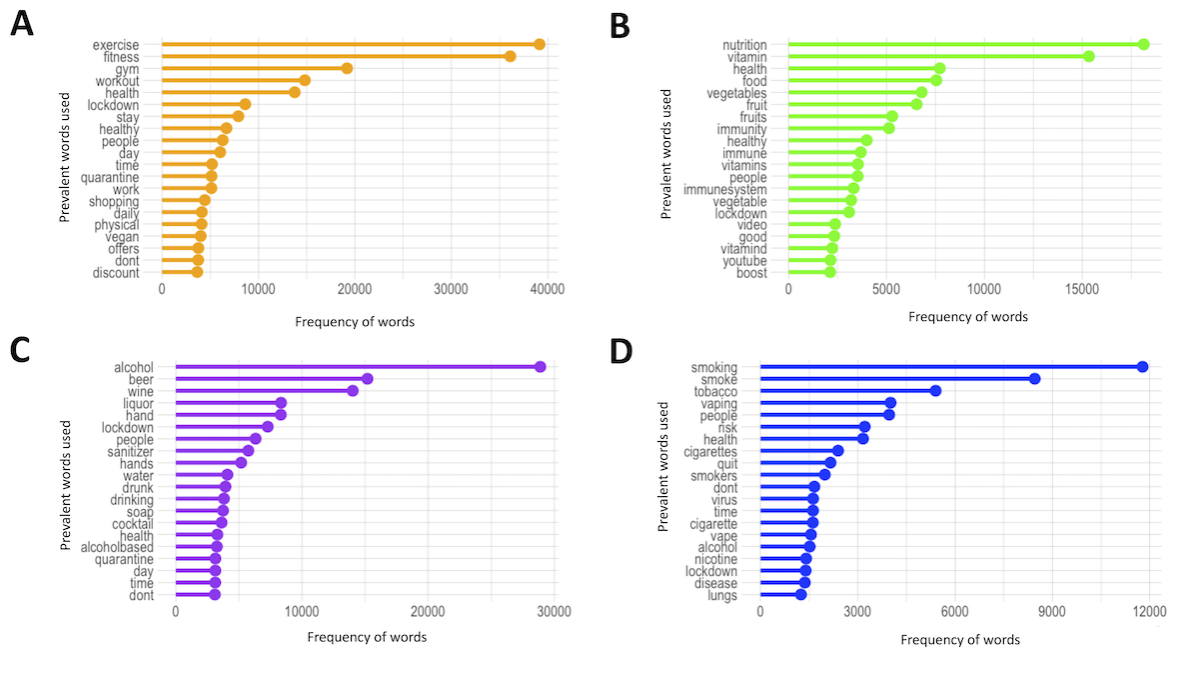
Frequency of the most prevalent words identified in each of the data sets (A) physical inactivity, (B) poor nutrition, (C) alcohol consumption, and (D) smoking.

**Figure 4:**
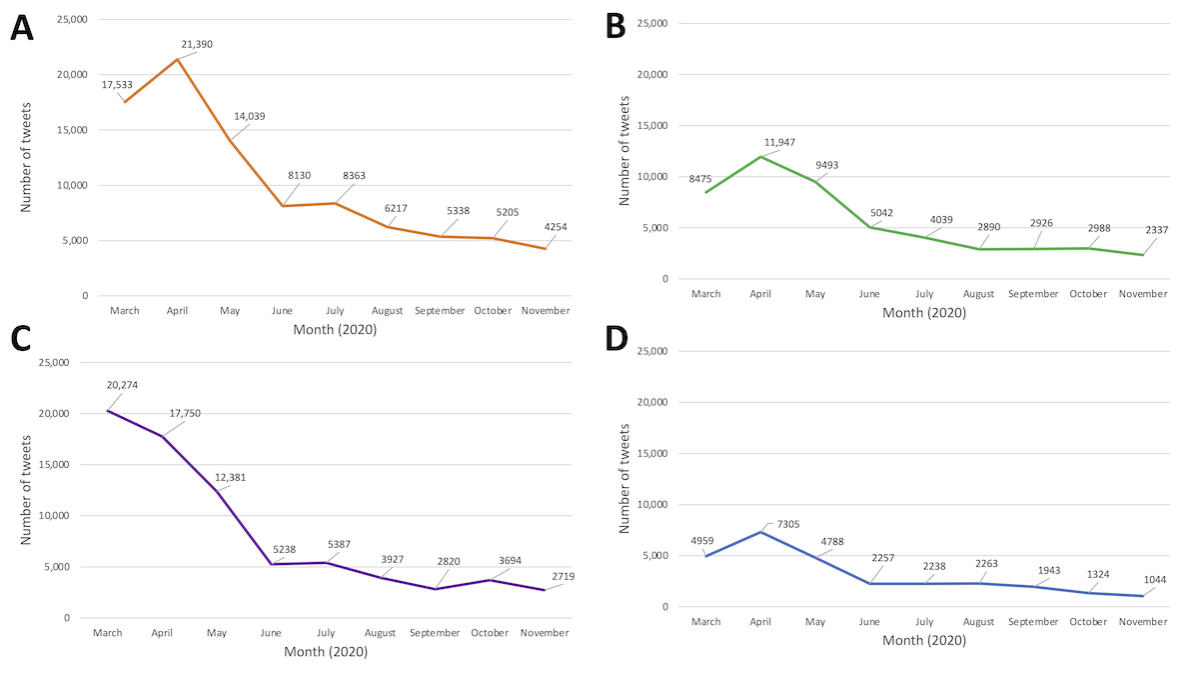
Frequency of cancer risk factor–related tweets between March 3 and November 30, 2020, displayed over monthly intervals (A) physical inactivity, (B) poor nutrition, (C) alcohol consumption, and (D) smoking.

The most common words used in the 4 data sets are shown in Figures 3A-3D. The frequency of cancer risk factor–related tweets between March 3 and November 30, 2020, are displayed over monthly intervals for each of the risk factors in Figures 4A-4D. Between May 2020 and November 2020, tweets peaked in April 2020, with the exception of the alcohol data set, which had a peak in March (Figures 4A-4D). After the peaks, there was a subsequent drop for the remaining time. The most prevalent words in the physical activity data set were *exercise*, *fitness*, *gym*, and *workout*, and the most prevalent words in the nutrition data set were *nutrition*, *vitamin*, *health*, and *food*. In the alcohol data set, the most common words were *alcohol*, *beer*, *wine*, and *liquor*, and in the smoking data set, the most common words were *smoking*, *smoke*, *tobacco*, and *vaping*.

### Sentiment Analysis

**Figure 5:**
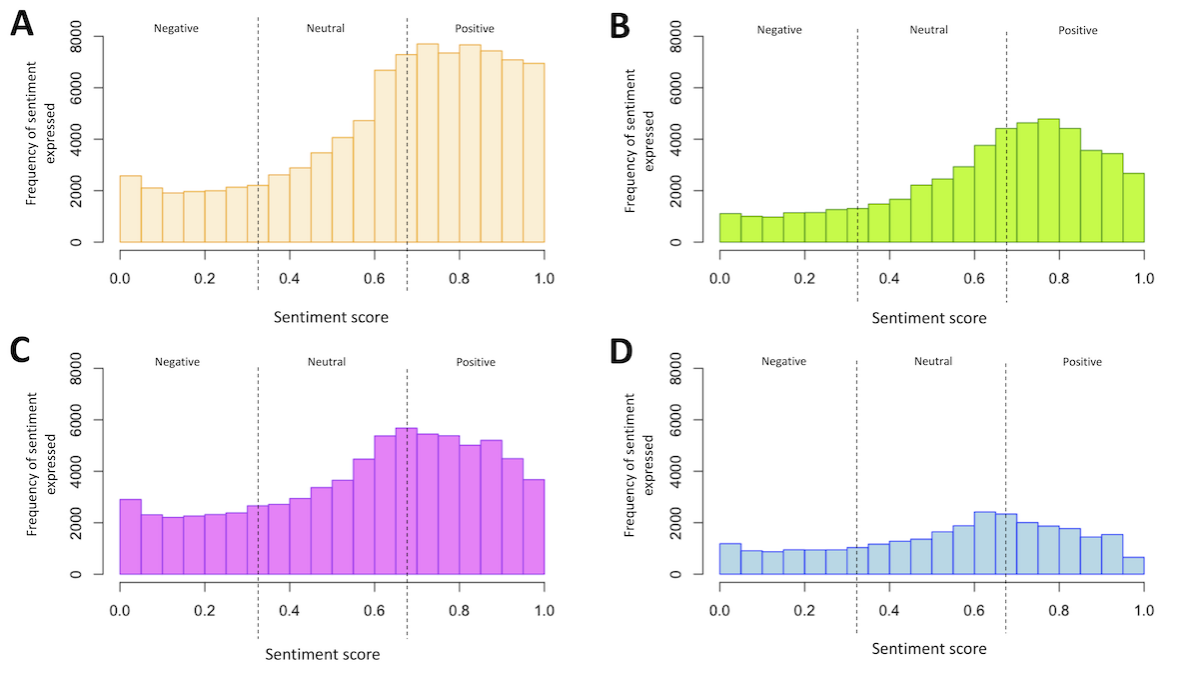
Distribution of sentiment scores for each of the risk factors between the values of 0 (negative) to 1 (positive); values between 0.35 and 0.65 are neutral (A) physical inactivity, (B) poor nutrition, (C) alcohol consumption, and (D) smoking.

The distribution of sentiment scores in each of the data sets is displayed in Figures 5A-5D. Table 2 summarizes the percentage and number of tweets classified as positive, neutral, or negative for each of the data sets.

**Table 2:** Percentage and number of tweets classified as either positive, neutral, or negative for each of the risk factors (N=243,913).

Risk factor	Positive tweets, n (%)	Neutral tweets, n (%)	Negative tweets, n (%)
Physical inactivity (n=90,813)	51,479 (56.69)	24,441 (26.91)	14,893 (16.4)
Poor nutrition (n=50,396)	27,939 (55.44)	14,507 (28.79)	7950 (15.78)
Alcohol consumption (n=74,484)	34,897 (46.85)	22,531 (30.25)	17,056 (22.9)
Smoking (n=28,220)	11,628 (41.2)	9753 (34.56)	6839 (24.23)

**Figure 6:**
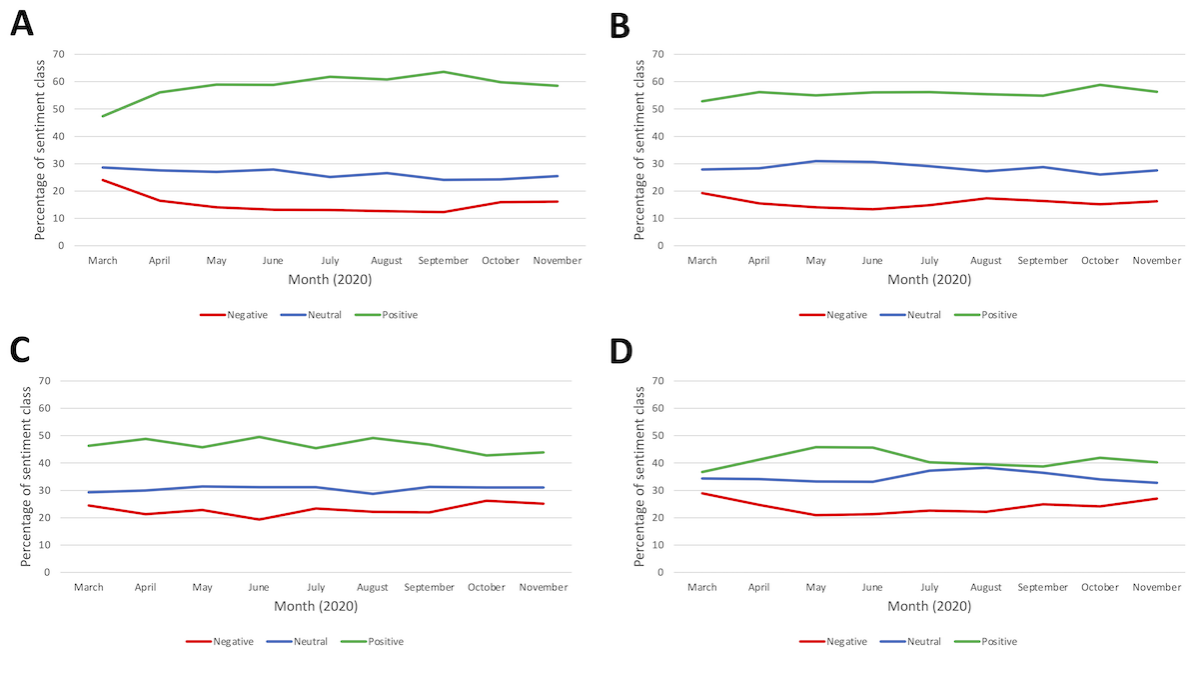
Changes in sentiment from March 3 to November 30, 2020, shown across the 9-month period for each risk factor (A) physical inactivity, (B) poor nutrition, (C) alcohol consumption, and (D) smoking.

Plots of positive, negative, and neutral sentiment for each of the 4 data sets by month are provided in Figures 6A-6D. The plots show that sentiments remained consistent.

On the basis of the results of the sentiment histogram and the sentiment displayed over time, most of the tweets about each risk factor were classified as having a positive sentiment. However, there was also a substantial proportion of negative and neutral tweets. Approximately 56.69% (51,479/90,813) of the tweets about physical activity were positive, 16.4% (14,893/90,813) of the tweets were negative, and 26.91% (24,441/90,813) of the tweets were neutral. Similar patterns were observed for nutrition, where 55.44% (27,939/50,396), 15.78% (7950/50,396), and 28.79% (14,507/50,396) of the tweets were classified as positive, negative, or neutral, respectively. For alcohol, the proportions of positive, negative, and neutral tweets were 46.85% (34,897/74,484), 22.9% (17,056/74,484), and 30.25% (22,531/74,484), respectively, and for smoking, the proportions were 41.2% (11,628/28,220), 24.23% (6839/28,220), and 34.56% (9753/28,220), respectively. The proportion of tweets that expressed each sentiment remained relatively stable and consistent over the study period.

### Emotion Analysis

The 8 emotions in the emotion analysis were analyzed by examining each word in the tweet and not the entire sentence in the tweet. Using this method, it was possible to distinguish the different emotions in each data set. Table 3 shows the percentage and number of tweets that were classified under each of the 8 emotions for each of the risk factors. The most represented emotion of each risk factor is italicized. Each tweet can be tagged more than once and falls under multiple emotion categories.

**Table 3:** Percentage and number of tweets classified under each of the 8 emotions for each of the risk factors.

Risk factors	Emotions, n (%)							
	Anger	Anticipation	Disgust	Fear	Joy	Sadness	Surprise	Trust
Physical inactivity (n=320,741)	21,696 (6.76)	61,186 (19.08)	14,138 (4.41)	45,929 (14.32)	50,478 (15.74)	33,551 (10.46)	24,268 (7.57)	*69,495 (21.67)* ^a^
Poor nutrition (n=176,564)	12,433 (7.04)	29,764 (16.86)	9045 (5.12)	26,798 (15.18)	26,836 (15.2)	18,532 (10.5)	10,832 (6.13)	*42,324 (23.97)*
Alcohol consumption (n=273,128)	28,233 (10.34)	39,158 (14.34)	16,492 (6.04)	41,848 (15.32)	*49,147 (17.99)*	36,481 (13.36)	16,390 (6)	45,379 (16.61)
Smoking (n=110,256)	10,404 (9.44)	17,564 (15.93)	9872 (8.95)	*23,066 (20.92)*	9504 (8.62)	7288 (6.61)	15,590 (14.14)	16,968 (15.39)

^a^Italicized values indicate the most represented emotion of each risk factor.

**Figure 7:**
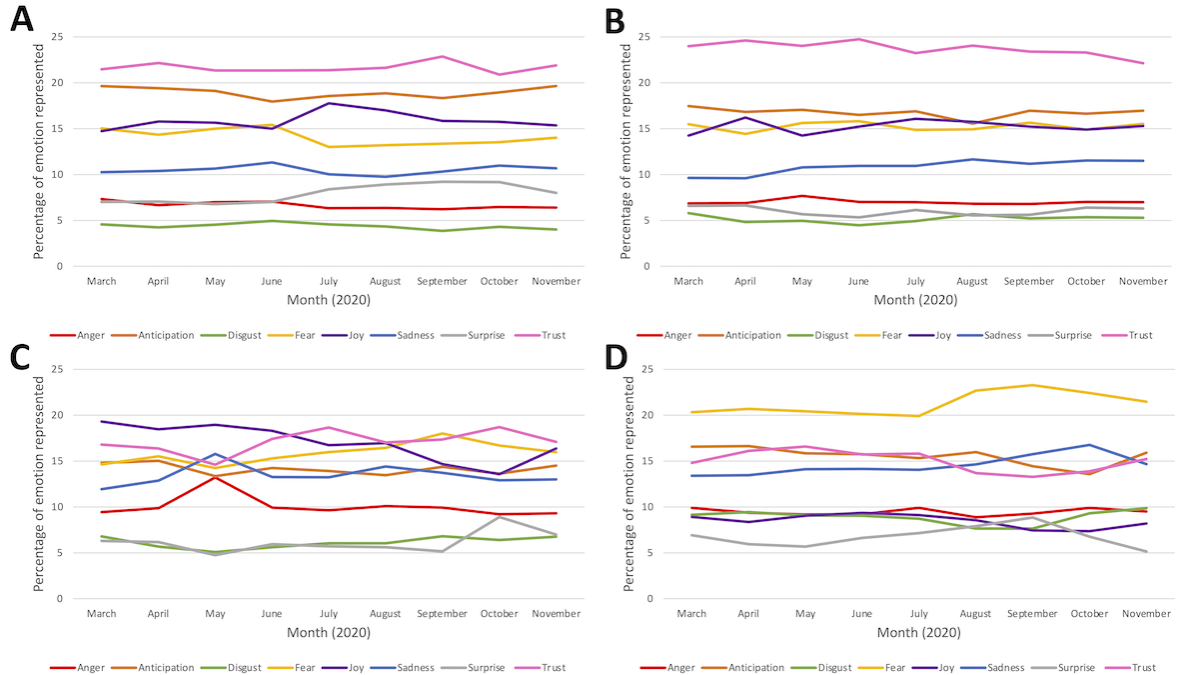
Changes in emotions represented from March 3 to November 30, 2020, shown across monthly intervals for each risk factor (A) physical inactivity, (B) poor nutrition, (C) alcohol consumption, and (D) smoking.

The represented emotions are displayed in Figures 7A-7D on a monthly basis for each of the 4 data sets.

In the physical activity and nutrition data set, the most common emotions were trust (69,495/320,741, 21.67% and 42,324/176,564, 23.97%, respectively) and anticipation (61,186/320,741, 19.08% and 29,764/176,564, 16.86%, respectively). In the alcohol data set, the most prevalent emotions were joy (49,147/273,128, 17.99%) and trust (45,379/273,128, 16.61%). In the smoking data set, the most common emotions were fear (23,066/110,256, 20.92%) and anticipation (17,564/110,256, 15.93%). On a monthly basis, the emotions seem to fluctuate more than the sentiment. In the physical activity data set, trust and anticipation stayed persistent over time. In the nutrition data set, trust remained the most common, followed by anticipation. In the alcohol data set, all the emotions fluctuated and were not consistent. In the smoking data set, fear was always the most commonly represented emotion, whereas the other emotions consistently overlapped as well.

## Discussion

### Principal Findings

The results of our study suggest that people were using Twitter to talk about modifiable cancer risk factors of interest during the first year of the pandemic. Our results show that it is feasible to use Twitter data to extract tweets relating to both COVID-19 and the 4 cancer risk factors. Our study demonstrated that unique sentiments and emotions in each of the 4 data sets could be captured. Furthermore, the results demonstrate that changes in sentiment and emotion can be identified over time.

Overall, the tweets about each risk factor had a positive sentiment and varied emotions across each data set. In the physical activity and nutrition data set, trust was the most represented emotion, whereas joy and fear were the most prevalent for the alcohol and smoking data sets, respectively. The number of tweets for each data set peaked from April to March 2020. After the respective peaks in the data sets, there was a subsequent drop in the number of tweets for the rest of the observed period. This might suggest that social media may be a timely source of data for public health surveillance during a public health emergency. This could be because during the pandemic, we were also combating an infodemic [50]. There was an overload of information on social media and other media platforms pertaining to health. This information either supported or contradicted the mainstream media. The drop of tweets after the peaks could also be derived as a pause in obtaining information through social media because of uncertainty of information, trustworthiness, or reliability. The most identified words suggest that many tweets were about the risk factors in relation to the context that we would expect (ie, in relation to health). However, in the alcohol data set, the terms *hand* and *hand sanitizer* frequently emerged suggesting that our search also inadvertently captured tweets about alcohol-based hand sanitizers. Similarly, in the smoking data set, although the most frequent words included *smoking*, *tobacco*, *vaping*, *risk*, *vape*, and *health*; words related to *fires* and *wildfires* also emerged suggesting that environmental smoke exposures were unintentionally captured.

Challenges of conducting a Twitter sentiment analysis include the algorithm and software misinterpreting the users’ intentions. Sarcasm is challenging and cannot be easily detected and understood by the machine [51]. In addition, it has been shown that marketing and promotion posts that use technical expressions are commonly mistaken as a positive sentiment [52]. Sentiment analysis has difficulties understanding mixed and differing opinions as well as cues that are not available in the lexicon or training data being used. In addition, during emotion analysis, a difficulty that is encountered is when a specific word in the tweet has 2 different meanings [53]. Similar or unrelated meanings of words can refer to different emotions or some words may cover multiple sentiments. There may have been tweets that were classified incorrectly when multiple sentiments were present. An example of a tweet with multiple sentiments is, “I’ll miss you sooo much! I can’t believe you have to leave...love you!” [53]. This tweet shows sentiments of both love and sadness. In the case of a tweet with more than one sentiment, it is difficult for the classifier to determine the dominant sentiment [53]. Tweets can also be misclassified by emotion as some of the emotions are closely related. However, a review and benchmark evaluation conducted by Zimbra et al [52] Evaluated Sentiment140 as a training classifier and found that it has performed well in previous Twitter sentiment analyses and is able to address the challenges that were faced when conducting these types of analyses. Sentiment140 uses a classifier with a high accuracy to recognize statements containing different opinions. For the emotion analysis, the NRC system was the top-ranked system in the 2013 Semantic Evaluation’s Sentiment Analysis in Twitter (SemEval SAT) competition based on its ability to contain 300,000 features for emotion lexicons [52]. These features include various sentiment lexicons, emotion lexicons, punctuation marks, emoticons, word length, and capitalization [52]. Therefore, the classifier and lexicon used in our study can overcome some of the challenges with Twitter sentiment and emotion analysis. When analyzing the sentiment and emotion analysis together, an example of an interpretation that can be drawn, specifically from the alcohol data set, is that alcohol from May to June 2020 brought people joy and that people were drinking more to forget about the pandemic. This, in turn, could potentially increase their risk for developing cancer. This conclusion can be made as the sentiment for the alcohol data set was positive and the most common emotion observed from this period was joy.

### Comparison With Prior Work

Previous sentiment or emotion analysis studies using Twitter data during the COVID-19 pandemic have investigated topics such as COVID-19 vaccines and the use of complementary, alternative, and integrative medicine [26,54,55]. Analyses of COVID-19 vaccine–related discussions show that the sentiment of the tweets was increasingly positive, and the most common emotion was trust followed by anticipation, which was also observed in our analysis of physical activity and nutrition tweets [54]. Another study that examined the public perception of the COVID-19 pandemic found that most tweets had negative sentiments, but the proportion of positive sentiment tweets increased as more information became accessible to the public, highlighting the importance of public health communication [55]. The emotion analysis of public perceptions showed that half of the tweets analyzed were defined primarily by the 3 emotions of fear, trust, and anticipation, which is similar to our results in tweets related to smoking [55]. Finally, a study examining complementary, alternative, and integrative medicine in the context of the COVID-19 pandemic reported similar results of an overall positive sentiment and the most predominant emotion of trust in the tweets [26]. In terms of behavioral cancer risk factors, our results are somewhat consistent with previous studies of smoking and physical activity that captured sentiment and changes during the pandemic period [22,23].

### Strengths

Our study had several strengths, including a large data set derived from tweets by many people worldwide at a time when traditional public health data collection (eg, surveys) was challenging. Although we conducted the analysis for this study a year later, these data were available in real time. We used a supervised machine learning approach to conduct both our sentiment and emotion analysis [38]. With this approach, we trained our analysis model using established approaches used in other studies of sentiment and emotion analysis that address some of the challenges in analyzing Twitter data. In addition, our research used person-generated content as a novel approach. This was an effective way to gather insights about public health issues, especially with the growing use of eHealth and mobile health technologies. By using social media and other web-based platforms, researchers and public health officials can reach a diverse audience, collect real-time data, and better understand how health issues affect different communities. Finally, we were able to observe trends and inconsistencies throughout the first 9 months from the beginning of the pandemic.

This study offers several implications for future research. The findings of this study prove that nontraditional data can continue to be used for public health research. Twitter data can be easily collected and provide valuable and real-time insights on sentiment and emotion of the general population. Public health policy makers, campaigns, or agencies can use such analyses for public health surveillance in the future. It can assist them by aiding them to prioritize their initiatives based on what people are tweeting about.

### Limitations

Our study was largely exploratory and had several limitations. First, we recognize that the Twitter data are not representative of the entire population and may not be generalizable. We used only English and original tweets, which further limits generalizability. Second, we cannot determine whether the context of all tweets was relevant for our purpose of understanding health risk factors. For example, we observed that in the alcohol data set when looking at the most frequent words tweeted, words such as *hand* and *sanitizer* were generated due to alcohol-based hand sanitizers. In addition, in the smoking data set, words such as *wildfire* were tweeted showing that within our cancer risk factor search, we collected tweets that were related to forest and wildfires. The unintended inclusion of wildfires could have resulted in the most common emotion being fear in the smoking data set as well. This is a limitation as the search terms used inadvertently identified other meanings of the words that were not related to health and cancer risk factors. Third, our selected search terms and use of a COVID-19 data set limited our search; however, given the scope of our study, we felt this was reasonable, but it may have resulted in missing other relevant tweets that did not use any of our keywords. The limitation to search terms could have introduced bias resulting in missed tweets. We used the Coronavirus tweet IDs made available by the George Washington University Libraries Dataverse that could be filtered instead of the Twitter API because of its ease of use and data availability. However, by using the Coronavirus tweet IDs as our data set, we were also limited to the data availability and could only include tweets from March 3 to December 3, 2020. Thus, we were unable to compare the data with a period before the pandemic. Fourth, sentiment and emotion analysis also have limitations as discussed earlier and although the results could theoretically be useful to understand how the public was feeling about cancer risk factors at the time of the study, it is challenging to interpret this in a way that is useful for public health surveillance. For example, although we observed that a large proportion of the tweets about alcohol were associated with emotions of fear, joy, and trust, we would not be able to readily draw conclusions about the changes in alcohol intake and whether there is a need for public health interventions.

### Future Work

Owing to the several limitations that did arise with the lack of specificity for search terms and the unintended inclusion of some tweets, future work is needed to understand the meaning of the results. Future studies should conduct a similar analysis on cancer risk factors before, during, and after the pandemic to further understand changes over time. Moreover, although it is advantageous to use social media data for health surveillance, a deeper dive via a qualitative approach combined with machine learning can also be taken to provide contextual examination of each written tweet. Combining qualitative analysis of the content of the tweets with the machine learning would allow for future researchers to understand the meaning behind specific language choices as well as social, cultural, or political factors that influence the content.

### Conclusions

In summary, the results of our study suggest that it is possible to measure changes in sentiment and emotions about cancer risk factors during a public health emergency using social media data. For all 4 risk factors, most tweets had a positive sentiment and varied emotions across the different data sets. Although there were limitations that arose owing to the lack of specificity, the findings of this study enable us to gain insights into both sentiment and emotion, which can provide timely and large-scale guidance for public health interventions or campaigns in the future.

## Abbreviations

API: application programming interface
NLP: natural language processing
NRC: National Research Council

## Data Availability

The Coronavirus tweet IDs data set is publicly available on the George Washington University Libraries Dataverse [27].

The coded data and filtered files are available upon reasonable request to the corresponding author.
